# Association of Serum Omentin-1 Concentration with the Content of Adipose Tissue and Glucose Tolerance in Subjects with Central Obesity

**DOI:** 10.3390/biomedicines11020331

**Published:** 2023-01-24

**Authors:** Marcelina Sperling, Teresa Grzelak, Marta Pelczyńska, Paweł Bogdański, Dorota Formanowicz, Krystyna Czyżewska

**Affiliations:** 1 Department of Medical Chemistry and Laboratory Medicine, Poznan University of Medical Sciences, 8 Rokietnicka Street, 61-701 Poznań, Poland; 2 Chair and Department of Physiology, Poznan University of Medical Sciences, 6 Święcickiego Street, 60-781 Poznań, Poland; 3 Chair and Department of Treatment of Obesity, Metabolic Disorders and Clinical Dietetics, Poznan University of Medical Sciences, 84 Szamarzewskiego Street, 60-569 Poznań, Poland; 4 Department of Nursing, Stanislaw Staszic State University of Applied Sciences in Pila, 10 Podchorążych Street, 64-920 Piła, Poland

**Keywords:** omentin-1, obesity, glucose tolerance, insulin resistance

## Abstract

Omentin is one of the few adipokines with potentially beneficial metabolic effects. The main aim of this study was to determine the association between serum omentin-1 levels and the occurrence of central obesity and abnormal glucose tolerance, taking into account gender. The study involved 88 participants aged 30–60, including 47 women and 41 men. Two subgroups among the obese subjects were distinguished—those with normal and abnormal glucose tolerance. Anthropometric and biochemical examinations and blood pressure measurements were performed. Omentin-1 concentrations were significantly lower among patients with obesity compared to those without obesity (*p* = 0.027) and, similarly, comparing men with abnormal glucose tolerance with men with normal glucose tolerance (*p* = 0.035). In contrast, no such pattern was observed in women. The multivariable regression model showed a significant effect of gender status and important factors of tissue insulin sensitivity, such as OGGT results, WHR and amount of body fat, on the variability of serum omentin-1 concentration in the entire study population (R^2^adj. = 13.7%; *p* = 0.003). High omentin-1 levels found in men with obesity and normal glucose tolerance suggest that omentin-1 protects against metabolic disorders associated with obesity in the male population.

## 1. Introduction

The excessive and abnormal accumulation of body fat leads to obesity, which has become a global epidemic threatening human health and life. Despite growing awareness of its negative health consequences, the number of obese people is steadily increasing. People with the abdominal type of obesity are particularly at risk of developing complications of excessive body weight. According to the recommendations of the IDF (The International Diabetes Federation) and the AHA/NHLBI (The American Heart Association and The National Heart, Lung, and Blood Institute), waist circumferences ≥ 80 cm for women and ≥94 cm for men are associated with an increased morbidity and mortality [1,2,3]. According to a May 2022 WHO report, waist circumference measurements, including the determination of waist-to-hip ratio, can be used interchangeably with the assessment of abdominal fat mass, which justifies the use of this measurement in the assessment of cardiodiabetes risk in obese patients [4].

Chronic positive energy balance, low physical activity and genetic predisposition lead to adipocyte hypertrophy and hyperplasia. This dysfunction, called adiposopathy or sick fat, negatively affects the paracrine and endocrine function and immune response of adipose tissue [2]. Obesity, especially of the central type, also disrupts the homeostasis of the secretion of adipokines, which are biologically active proteins secreted by adipocytes. Adipokines have several important functions in the human body, including regulating metabolic and inflammatory processes and adipogenesis [5].

Omentin (known as intelectin, intestinal lactoferrin receptor, endothelian lectin HL-1, galactofuranose-binding lectin) is an adipokine whose gene is expressed mainly in stromal vascular fraction cells of adipose tissue, rather than subcutaneous [6]. It has two isoforms, omentin-1 and omentin-2. Omentin-1 is the main form circulating in human blood [7]. The omentin gene is located in a chromosomal region (1q21.3) associated with type 2 diabetes [8]. This adipokine plays a role in the development of inflammatory diseases. It has beneficial effects on energy homeostasis, glucose metabolism, cardiovascular system functioning and the reduction of oxidative stress [9,10]. In addition, omentin-1 shows protective effects against cancer, atherosclerosis, and metabolic bone disease [11].

Analyses of the relationship between serum omentin-1 concentrations and anthropometric parameters have not provided clear conclusions. Initial studies mainly provided evidence that the concentration of omentin-1 decreases with increasing body weight [12]. However, some reports have suggested the existence of inverse relationships between its concentration and the presence of overweight or obesity [13,14,15].

Conducting further studies evaluating omentin concentrations in different population groups is therefore justifiable and may help to understand its functions and mechanism of action. This is particularly important because omentin-1 appears to be one of the few adipokines with potentially beneficial metabolic effects, and the factors regulating omentin gene expression in adipose tissue still need to be better understood.

In the present study, we evaluated and compared omentin concentrations in a group of centrally obese patients, including those with normal as well as newly diagnosed abnormal glucose tolerance. Presumably, the degree of insulin resistance, glucose metabolism and the occurrence of obesity complications are related to omentin serum concentrations. Thus, the research hypothesis that patients with central obesity of various degrees correlated with abnormal glucose tolerance, compared to obese subjects with normal tissue insulin sensitivity, will have lower serum concentrations of omentin was established.

The study’s main aim was to determine the relationship between serum omentin concentration and the incidence of central obesity and impaired glucose tolerance, taking into account the gender of the subjects.

## 2. Materials and Methods

### 2.1. Inclusion and Exclusion Criteria

Qualification for the study and control groups was based on an analysis of available medical documentation and medical and dietary interviews. They took into account past and current medical conditions, as well as medications and dietary supplements taken, diet and level of physical activity. Inclusion criteria for the study group were BMI (Body Mass Index) > 30 kg/m^2^, the waist circumference of women ≥ 88 cm and men ≥ 102 cm, and WHR (Waist–Hip Ratio) of women ≥ 0.80 and men ≥ 1.00. This allowed us to qualify individuals with the highest cardiodiabetes risk associated with obesity. Volunteers in the control group did not meet any of the mentioned conditions.

Exclusion criteria for the study were retrospective and included previously diagnosed and treated diabetes, impaired glucose tolerance or abnormal fasting blood glucose. In addition, chronic disease of the endocrine glands, liver, kidney and pancreas, and the presence (currently and in the five years preceding the study) of cancer or cardiovascular incidents. Study participants with pre-existing dyslipidemia and/or hypertension continued to take the necessary medications. Analyses were performed on subjects who did not report bad mood or symptoms indicative of acute infection on the day of the study. According to the subjects’ declarations, their body weight was stable in the three months preceding the survey. None of them played sports competitively, and their physical activity was low or moderate. Participants did not work in shifts. Eating habits were verified by analysing three-day food diaries. Analysis of the dietary data allowed us to exclude from the study people whose eating habits were unconventional or who followed ‘alternative diets’, e.g., vegetarianism or a ketogenic diet which could affect omentin blood concentrations. An additional exclusion criterion was pregnancy and lactation in the present and in the previous six months.

### 2.2. Study Population

**Figure 1:**
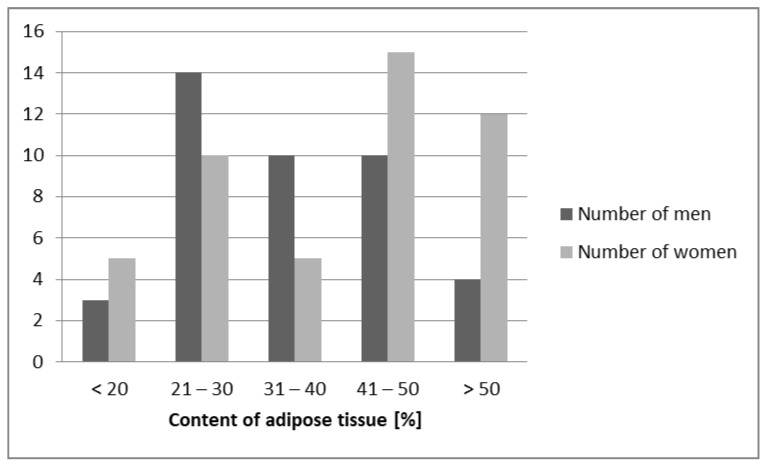
Characteristics of the study population in terms of content of adipose tissue [%] in the male and female groups.

The study included 88 subjects aged 30–60 years, including 47 women and 41 men. The mean ± SD age of the study population was 45.22 ± 8.08 years. The study group (*n* = 51), consisting of 27 women (47.71 ± 8.12 years) and 24 men (46.83 ± 8.01 years) with obesity (see Figure 1), was divided into two subgroups based on the results of the 75 g Oral Glucose Tolerance Test (OGTT) as recommended by the World Health Organization and the International Diabetes Federation [16]:

- Subjects with obesity and normal glucose tolerance (*n* = 24) (glycemia at 120 min OGTT < 7.8 mmol/L)—NGT (Normal Glucose Tolerance),
- Subjects with obesity and impaired glucose tolerance (*n* = 27) (glycemia at 120 min OGTT ≥ 7.8 mmol/L)—AGT (Abnormal Glucose Tolerance).

The control group included 37 volunteers without obesity, including 20 women (45.3 ± 8.21 years) and 17 men (43.29 ± 6.44 years). The control group consisted of volunteers who applied for the study in response to the advertisement and met the conditions: age 30–60 years, BMI < 30 kg/m^2^ and >18.5 kg/m^2^; waist circumference of women < 88 cm and men < 102 cm and WHR of women < 0.80 and men < 1.00. Recruitment for the study and control groups lasted one year and was conducted simultaneously.

Anthropometric examinations on an empty stomach in the morning were performed. Measurements were taken only in underwear. The study participants were informed that 24 h before the test they should not consume alcohol and energy drinks, coffee or tea and should not exercise. Height and weight were estimated using a certified electronic scale (SECA 285 Wireless, Hamburg, Germany). The circumferences of the waist (midway between the lower edge of the ribcage arch and the upper iliac crest) and hips (at the height of the greater ileum) were measured with an accuracy of 0.5 cm. The results were used to calculate BMI (Body Mass Index) and WHR (Waist–Height Ratio). Body composition analysis was conducted using the electrical bioimpedance method using a Bodystat 1500 analyzer (Bodystat Ltd., Isle of Man, UK). In accordance with the producer’s recommendations, the test was performed in the lying position using data on the subject’s sex, height and weight and physical activity level. The electrical bioimpedance method used required the placement of 4 electrodes on the patients’ hands and feet and allowed the determination of body fat and lean body mass, i.e., total muscle mass and water content. The essence of the BIA method is to measure the resistance of a low-intensity electrical current (≤0.1 mA) passed through tissues. Study participants were informed that 24 h before the test they should not consume alcohol or energy drinks, coffee, tea or carry out intense exercise.

### 2.3. Blood Pressure Measurement

Before starting the study, participants were asked to assume a sitting position and rest for 5 min. Systolic and diastolic blood pressure measurements were taken three times at two-minute intervals, and the results were averaged according to the recommendations of the European Society of Hypertension and the European Society of Cardiology [17] using an accredited sphygmomanometer (model 705IT, Omron Corporation, Kyoto, Japan).

### 2.4. Biochemical Tests

Blood samples were collected in the morning, while fasting. Most of the analyses, i.e., assessment of glucose, triglycerides, total and HDL-C cholesterol, and insulin, were performed immediately. Due to the methodology, determining adipokine concentrations required centrifugation of serum and then freezing at −80 °C. Assessment of serum glucose in venous blood was performed by the enzymatic method with hexokinase, using a Cobas c analyser from Roche Diagnostic GmbH (Roche Diagnostic, Mannheim, Germany). Insulinemia was assessed using immunochemical tests according to Chemiflex protocols (Abbott Diagnostics, Lake Forest, IL, USA). The results of insulin and fasting glucose concentrations were used to calculate an index of insulin resistance: HOMA-IR (Homeostasis Model Assessment—Insulin Resistance): HOMA-IR = glycemia (mmol/L) × fasting insulin (mU/L)/22.5.

Concentrations of triglycerides, total cholesterol, and HDL-C (High-Density Lipoprotein Cholesterol) were measured by commercial assays using colourimetric enzymatic methods. Since triglyceridemia was less than 400 mg/dL (4.5 mmol/L), the Friedewald formula [18] was used to calculate LDL-C (Low-Density Lipoprotein Cholesterol).

The concentration of omentin-1 was measured by using a certified immunoenzymatic (Omentin (human) EIA Kit (Cayman Chemical Company, Ann Arbor, MI, USA), strictly following the manufacturer’s recommendations. The read absorbance of the standards on an MR-96 plate reader from CLINDIAG SYSTEMS B.V.B.A. (Pollare, Belgium) made it possible in each case to plot a 4-parameter standard curve (using Sigma Plot 11.00 software) and determine serum protein concentrations.

### 2.5. Statistical Analyses

Statistical analyses of the results were performed using MS Excel, STATISTICA 13 (StatSoft Inc., Tulsa, OK, USA) and SPSS Statistics (IBM Corp., Armonk, NY, USA). The normality of the data distribution for small groups was determined using the Shapiro–Wilk test and for large groups using the Kolmogorov–Smirnov test. When the conditions of normal distribution and homogeneity of variance (according to Levene’s test) were met for quantitative variables, the Student’s t-test was used. The non-parametric Mann–Whitney U test was used for data not meeting the normal distribution condition. When differences in variance were found for data meeting the conditions of normal distribution, the Welch test was used. Pearson’s and Spearman’s tests were used to analyse the correlation of the studied data. The results of all statistical analyses were considered statistically significant when the significance level was *p* < 0.05. The effect of the variables on omentin-1 concentrations was analysed using multivariable regression models. A multivariable regression model included all analysed parameters with a bivariate *p* < 0.25. Moreover, the effect of the variables on omentin-1 values (above the median value vs. median value and below) was analysed using logistic regression models. The fit of the logistic regression models was assessed using the likelihood ratio chi-square test (LRT) and the Hosmer–Lemeshow goodness-of-fit test [19].

## 3. Results

### 3.1. Anthropometric Studies

**Figure 2:**
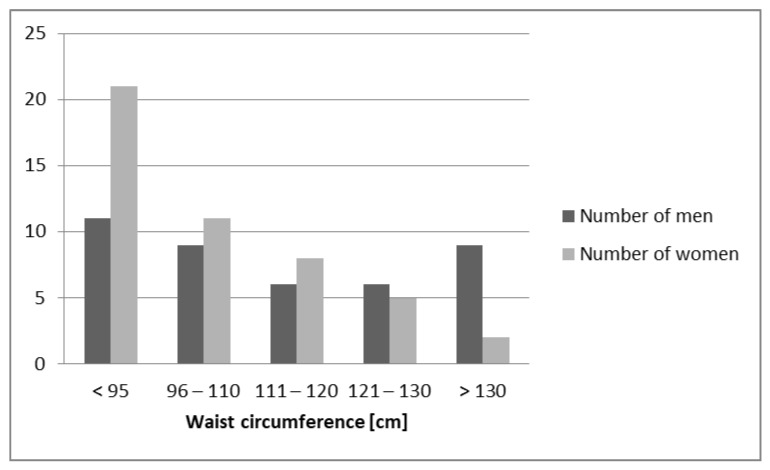
Characteristics of the study population, including waist circumference, in the male and female groups.

The study population (*n* = 88) included individuals with different adipose tissue content and various waist circumferences (Figure 1 and Figure 2). Following the objectives of the conducted research, comparing the mean ± SD results of anthropometric tests conducted in the study and control groups, there were statistically significant differences in body weight and waist and hip circumferences, as well as BMI and WHR calculated from the obtained data. In addition, a higher content of fat mass [%] and lower content of lean body mass [%] were shown in the study group, compared to the control group. However, there were no differences in the height of the subjects. Patients with central obesity and normal glucose tolerance and obese patients with abnormal glucose tolerance did not differ in anthropometric parameters (Table 1). The BMI range in the control group was 19–28.7 kg/m^2^, while in the study group it was 31.1–51.6 kg/m^2^, and the mean BMI values, according to the study design, were statistically significantly different (*p* < 0.0001). In addition, it is worth looking at the difference in waist circumference and WHR values; in the study group they were: waist circumference in women (range 93–140 cm); men (range 110–160 cm); WHR: in women (range 0.86–1.00); men (range 1.03–1.14) and in the control group waist circumference in women (range 63–84 cm); men (range 82–97 cm); WHR: in women (range 0.71–0.79); men (range 0.85–0.92). In our assessment, the control group was selected according to the study design.

### 3.2. Metabolic Studies

**Figure 3:**
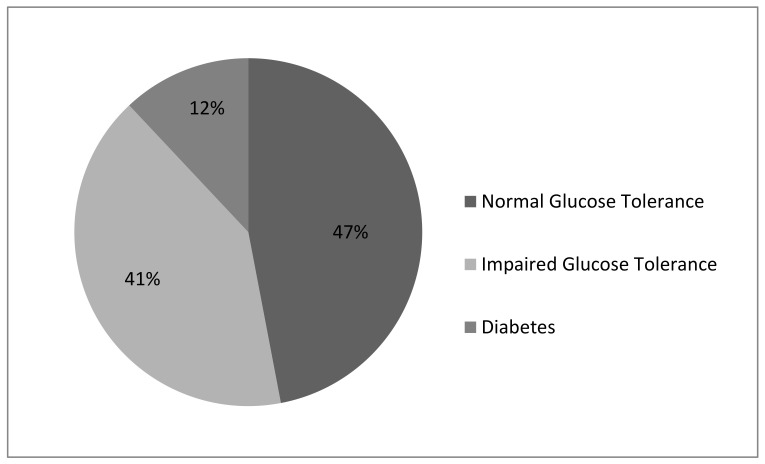
Interpretation at 120 min of the Oral Glucose Tolerance Test in the investigated group (obese subjects). Normal Glucose Tolerance: glycaemia < 140 mg/dL (7.8 mmol/L); Impaired Glucose Tolerance: glycaemia 140–199 mg/dL (7.8–11.0 mmol/L); Diabetes: glycaemia ≥ 200 mg/dL (11.1 mmol/L).

More than half of the obese group was diagnosed with abnormal glucose metabolism—impaired glucose tolerance or diabetes (Figure 3).

The results of the carbohydrate metabolism studies indicated differences in fasting glucose and insulin concentrations among the groups analysed. The HOMA-IR tissue insulin resistance index, calculated from these, was significantly higher among obese than lean subjects. Differences were also found in HDL-C and triglyceride concentrations, while LDL-C and total cholesterol concentrations were comparable in both groups (Table 2). Individuals with central obesity had significantly lower serum omentin-1 concentrations than the control group.

Comparing the results of subjects with obesity and NGT and patients with obesity and AGT, there was a marked difference in median insulinaemia and HOMA-IR values and systolic blood pressure values. No differences were observed for lipid metabolism parameters. Moreover, there was a trend towards higher omentin concentrations in people with NGT compared to those with AGT (Table 3).

There was no statistically significant difference in omentin-1 (ng/mL) serum concentrations between men and women (256.77 ± 123.47 vs. 256.15 ± 95.0) in the overall population and the subgroups with and without obesity. On the other hand, omentin-1 concentrations were slightly lower in women and men with obesity compared to representatives of the same sex without obesity. However, these differences were not statistically significant (Table 4).

Among patients with obesity and NGT, significantly higher omentin-1 concentrations were observed in men than in women. Similar patterns were not found among those with obesity and AGT. In another comparative analysis conducted in the obese group, gender was the main criterion for division. On this basis, men with AGT had lower serum omentin-1 concentrations (Table 5).

### 3.3. Correlation Analyses

Correlations between omentin-1 concentrations and anthropometric and metabolic parameters were analysed for the whole studied population, and separate analyses were performed for selected groups and subgroups. In the entire studied population (*n* = 88), we found that omentin-1 concentration correlated negatively with body weight (r_p_ = −0.240; *p* = 0.024), BMI (r_p_= −0.227; *p* = 0.033) and content of fat mass (r_p_ = −0.224; *p* = 0.036).

In the investigated group, negative correlations between omentin-1 concentration and body weight and BMI were only shown in obese women. In contrast, no significant correlations between omentin-1 concentration and the assessed anthropometric parameters were found in the control group. In obese men, a negative correlation between omentin concentration and glycemia in the second hour of the oral glucose tolerance test was observed. In the control group and among non-obese women, a positive relationship between omentin concentration and fasting insulinaemia was found. In the present study, there were no significant correlations between omentin-1 concentrations and lipid metabolism exponents or blood pressure values in the entire studied population, or separately in the investigation and control groups (Table 6).

### 3.4. Regression-Based Analyses

The multivariable regression model showed a significant effect of results in oral glucose test, WHR and fat mass [kg] on the variability of serum values of omentin-1 in research subjects (*p* = 0.007, R^2^adj. (adjusted R-squared) = 10.2%) (Table 7). To describe this phenomenon more precisely, an additional variable was included in the model, namely gender status. Adding gender status to the multivariable model allowed us to explain the variation in omentin-1 better: up to 13.7% (*p* = 0.003).

According to the logistic regression model, lowering body weight by every 1 kg in the men surveyed (*n* = 41) increases the chance of omentin-1 values above the median by 3% (LRT = −25.535, *p* = 0.019). As the Hosmer–Lemeshow statistic was 4.991 (*p* = 0.800), therefore, the model was judged to fit the observed data well [19]. Lowering body weight by every 1 kg in the women surveyed (*n* = 47) increases the chance of omentin-1 values above the median by 2.5%, according to the logistic regression model. As the *p*-value was relatively low (*p* = 0.209) in the Hosmer–Lemeshow test and the value of R^2^ Nagelkerke was equal to 0.120 in the case of women, the model was judged to not fit the observed data so well as in the case of men (Table 8).

## 4. Discussion

Omentin may be particularly important in the prevention of metabolic diseases associated with central obesity. Reduced omentin levels are associated with an increased risk of health complications of excess body weight, particularly type 2 diabetes [20]. To our knowledge, there are no studies on the concentrations of this adipokine in pre-diabetic women. Searching Pubmed and Cochrane databases, the authors arrived at analyses including only men with this condition [21]. Analyses of the concentrations of adipokines with potential beneficial effects on glucose metabolism in patients with central obesity of varying severity may help to understand the phenomenon of “healthy obese”. In our study, we found that people with central obesity have lower serum omentin-1 concentrations compared to the non-obese group, and a decrease in serum omentin-1 concentrations accompanies the development of abnormal glucose tolerance. Correlations between omentin-1 concentrations and anthropometric parameters and exponents of glucose metabolism were demonstrated. This is an important observation because it applies to people with new diagnoses and not-yet-treated glucose tolerance disorders—impaired glucose tolerance and type 2 diabetes. Based on studies, including our own, it can be speculated that maintaining high concentrations of omentin may protect against abnormal glucose tolerance.

Many studies evaluating the relationship between omentin concentrations and anthropometric parameters have provided conflicting results. Some have shown that omentin concentrations increased after a low-calorie diet [22,23], and others have shown that reduction diets decrease concentrations of this adipokine [24,25]. Auguet et al. showed that omentin gene expression in visceral adipose tissue was significantly lower in women with extreme obesity compared to lean female subjects. In addition, they found significantly lower serum omentin concentrations in obese women than in normal-weight women [12]. In contrast, Derosa et al. assessed omentin levels in patients with obesity compared to those without obesity and found no statistically significant differences between the two groups [26]. A comparison of omentin concentrations in the serum of women with and without obesity showed no differences. At the same time, it is worth noting that the female gender physiologically has a higher percentage of adipose tissue than the male gender. Negative correlations were also found between serum omentin concentrations and body weight, BMI, and fat mass content [%]. The first two correlations were particularly noticeable among obese women.

It is thought that reduced omentin concentrations may promote the development of insulin resistance, but the mechanism of this process still needs to be fully elucidated [11,27]. In vitro studies have shown that omentin increases tissue sensitivity to insulin. It stimulates protein kinase B (Akt) phosphorylation, enhancing insulin signal transduction and increasing insulin-dependent glucose transport in isolated human adipocytes. Omentin also stimulates AMP-activated protein kinase B (AMPK), inhibiting gluconeogenesis and a fall in blood glucose levels. The adipokine in question also can bind to lactoferrin, a protein implicated in the occurrence of insulin resistance. The anti-inflammatory properties of omentin have also been demonstrated. These most likely involve the inhibition of TNF-α-induced vascular cell adhesion molecule 1 (VCAM-1) gene expression in smooth muscle cells by preventing the activation of p38 protein and Jun *n*-terminal Kinase (JNK) [27,28,29]. It has been speculated that the level of omentin messenger RNA (mRNA) expression in the epicardium may influence the development and course of ischaemic heart disease. It has been shown that, in patients with type 2 diabetes, omentin mRNA expression in epicardial adipose tissue is lower than in healthy individuals. Relative to the above scientific reports, it can be concluded that omentin is a molecule associated with the development and course of obesity, as well as being involved in glucose metabolism and tissue insulin sensitivity [10,28,29].

Studies on the relationship between omentin levels and the occurrence of impaired glucose tolerance in humans have given conflicting results [27]. Most scientific evidence shows reduced omentin levels in patients with glucose metabolism disorders, such as diabetes type 1 and type 2, impaired glucose tolerance, and gestational diabetes, compared to healthy individuals [30,31,32,33]. However, there are also reports of higher levels of this adipokine in patients with diabetes compared to those without diabetes. An example is a Flehmig et al. study comparing omentin levels among obese patients with Diabetes Mellitus Type 2 (DM2) and obese populations without DM2 [34]. Similarly, Elsaid et al. showed higher mean omentin concentrations in obese women with diabetes compared to lean, healthy women [35]. Interesting results by Polish researchers (Komosińska-Vassev et al.) were presented. They assessed omentin concentrations in obese individuals diagnosed with type 2 diabetes before the introduction of insulin treatment and after six months of therapy. At baseline, omentin concentrations were higher in obese patients with DM2 than in the control population, which consisted of obese subjects with normal glucose metabolism. A significant increase in omentin concentrations was also observed as a result of insulin therapy. Thus, it has been suggested that the applied treatment in diabetic patients may be responsible for the differential results in concentrations of this adipokine [36]. It is worth noting that studies on the correlation between omentin concentrations and specific exponents of glucose metabolism and tissue insulin sensitivity are also inconclusive. Most researchers found a negative correlation between the level of omentin gene expression in visceral adipose tissue cells and blood concentration versus fasting glycaemia, HOMA-IR, and fasting insulinaemia [37,38,39,40]. On the other hand, Urbanova et al. showed no significant association between omentin levels and fasting glycaemia in patients with morbid obesity and type 2 diabetes [41]. Similarly, Catoi et al. found no significant correlation between omentin levels and fasting blood glucose in patients with giant obesity [42]. Additionally, Hossein-Nezhad et al. showed no association between omentin levels and any indicator of tissue insulin resistance [43]. In our study, a trend towards higher serum omentin concentrations was found in subjects with normal glucose metabolism, as reported by most investigators. This was statistically significant among men in this subgroup. In addition, a correlation was found between omentin concentrations to fasting insulin values in the non-obese group (particularly in women) and glycemia at 120 min of the OGTT in the group of men with obesity. In contrast, no correlation was observed between the concentration of the adipokine in question and HOMA-IR values in either group.

The conflicting results of previous studies indicate that multiple factors may influence omentin concentrations and that the mechanisms regulating omentin synthesis are complicated. It has been proven that insulin and glucose significantly reduce gene expression and omentin production in network adipose tissue. Therefore, hyperinsulinaemia leads to a significant reduction in circulating omentin concentrations in healthy individuals. Insulin and glucose play a direct or indirect role in regulating the synthesis of this adipokine. Further studies are needed to understand the factors influencing omentin synthesis and secretion in subjects with normal and abnormal glucose tolerance.

A consequence of impaired glucose metabolism and insulin resistance is cardiovascular disease. Several studies have found reduced omentin levels in patients with coronary heart disease or ischaemic stroke [28,44,45]. Presumably, omentin may reduce the risk of vascular complications in obese and diabetic patients. According to some researchers, a decrease in levels of omentin is an independent predictor of coronary heart disease and is associated with the course of this condition [8]. In a different view, Niersmann et al. evaluated the risk of cardiovascular incidents in patients with type 2 diabetes. They showed that higher levels of omentin are associated with an increased risk of myocardial infarction, stroke and death from cardiovascular causes in people with diabetes [46]. Similar conclusions were reached by Wittenbecher et al. They found no evidence for a protective role of omentin against diabetes type 2. They even suggested an increased risk of DM2 associated with high omentin concentrations among study participants [47]. Given the above, omentin affects cardiovascular risk. The increase in its concentration in people with complications of obesity and diabetes observed by some researchers may indicate some compensatory mechanism of its secretion, which, however, may be insufficient in some cases.

Previous studies have shown that levels of omentin are associated with macroangiopathic complications in patients with type 2 diabetes [8,28,45]. It is possible to observe confident relationships between glucose levels and lipid management parameters. For example, a positive correlation was found between omentin concentrations and HDL levels and a negative correlation with triglyceridaemia in studied men with significant stenosis (greater than 75%) in at least one major coronary artery [48]. In our study, no association was observed between omentin-1 levels and total cholesterol, LDL, HDL, and triglycerides. It should be noted that some of the subjects with central obesity were taking medication to control dyslipidaemia. There is scientific evidence to suggest that specific pharmacological agents, such as statins, may raise omentin concentrations, although, to date, their mechanism of action has not been determined [49].

Omentin-1 may also be involved in blood pressure control. Evidence from experimental and clinical studies suggests an effect of this adipokine on vascular reactivity and atherosclerosis. Omentin increases endothelial nitric oxide synthesis and stimulates revascularisation after ischaemic incidents. In addition, omentin can inhibit the secretion of TNFα and other pro-inflammatory cytokines in vascular endothelial cells [50,51]. However, in our study, no association was observed between omentin levels and blood pressure values. Aliasghari et al., in patients with non-alcoholic fatty liver disease and tissue insulin resistance, showed a marginally significant association between omentin levels and systolic blood pressure and no association with diastolic blood pressure values [52]. Other researchers who have observed an association between the levels of the adipokine in question and blood pressure values have suggested that regular assessment of blood levels of omentin may help assess the effectiveness of insulin therapy and, therefore, the risk of developing vascular disorders [36]. The lack of significant correlations between omentin-1 concentrations and blood pressure values in our study can be explained by the fact that the majority of centrally obese subjects were using hypertension medication.

The occurrence of gender-dependent differences in omentin secretion is controversial. On the one hand, De Souza Batista et al. [53] observed that slim women have higher omentin concentrations than slim men. Additionally, on the other hand, Moreno-Navarrete et al. showed the opposite pattern. Assessing the effect of a four-month reduction diet on concentrations of this adipokine in obese subjects, they observed that it was higher in the male group than in the female group. After the completion of the experiment, omentin concentrations increased proportionally in both groups [22]. Moreover, higher concentrations of omentin were noted in rats who were given oestrogen [54]. Borowski et al. showed no correlations between omentin and oestradiol or testosterone levels in patients with prostate cancer and associations with sex steroids and metabolic syndrome. However, they have demonstrated negative associations between bioactive testosterone, calculated as testosterone/SHBG ratio, and omentin, and a positive correlation between SHBG (sex hormone-binding globulin) and omentin level. SHBG is a carrier protein which is responsible for the transport of sex hormones to their target cells, so it determines their biological activity. SHBG levels are affected by insulin, nutritional and metabolic factors. One of the most important regulators of SHBG is hepatocyte nuclear factor 4 alpha (HNF4-α), a member of the nuclear receptor family responsible for regulating many genes engaged in glucose, cholesterol, and fatty acid metabolism [55].

In our study, significantly higher serum concentrations of omentin-1 were observed in obese men with NGT compared to women from this group. The multivariable regression model showed a significant effect of gender status and important factors of tissue insulin sensitivity such OGGT results, WHR and amount of body fat (kg) on the variability of serum levels of omentin-1 in the case of the entire study population. The multivariable regression model, including four parameters (WHR, OGGT results, amount of body fat (kg) and gender status), explained more per cent of the variation in results of omentin-1 concentration (R^2^adj. = 13.7% and *p* = 0.003) than a multivariable regression model without gender status (WHR, OGGT results, amount of body fat (kg); R^2^adj. = 10.2% and *p* = 0.007). According to logistic regression, in men, compared to women, lower body weight values were associated with a higher chance of omentin-1 concentrations above the median, i.e., the occurrence of values higher than 241.15 ng/mL. Presumably, serum omentin-1 concentrations may depend not only on the content of body fat, WHR and glucose tolerance status, which can be modified by exercise and diet, but also on gender [41].

### Limitations

The presented study has some limitations. The investigated and control groups were carefully selected to be compared, but the sample size, due to exclusion criteria, was relatively small. The obese patients were on necessary pharmacotherapy, which may have affected their metabolic balances and omentin-1 concentrations.

## 5. Conclusions

Our study shows that omentin-1 concentrations decrease with increasing body weight, BMI, and body fat content. Contrary to this trend, surprisingly high blood concentrations of omentin-1 were observed in men with obesity and normal glucose tolerance. Perhaps the increase in blood concentrations of omentin-1 can be linked to the occurrence of the ‘healthy obese’ phenomenon. In addition, the obese group with normal glucose tolerance showed significantly lower omentin-1 concentrations among women than men. A significant effect of gender status and important factors of tissue insulin sensitivity such OGGT results, WHR and amount of body fat on the variability of serum concentration of omentin-1 in the entire study population argues that gender is an important (and often overlooked) factor affecting the synthesis of this adipokine. The search for therapeutic strategies to raise omentin-1 levels may, in the future, contribute to reducing the risk of obesity-related glucose metabolism disorders.

## Figures and Tables

**Table 1:** Anthropometric characteristics of the study population.

Parameter	Investigated Group Obesity (*n* = 51)			Control Group no Obesity (*n* = 37)	*p* **
	Normal Glucose Tolerance (NGT) (*n* = 24)	Abnormal Glucose Tolerance (AGT) (*n* = 27)	*p* *		
Body weight (kg)	112.71 ± 24.44	115.37 ± 24.38	0.699	72.24 ± 14.778	**<0.001**
Body height (cm)	172.25 ± 9.87	170.89 ± 10.02	0.628	171.0 ± 10.121	0.806
BMI (kg/m^2^)	37.71 ± 5.98	39.34 ± 6.38	0.352	24.47 ± 3.074	**<0.001**
Waist circumference (cm)	117.88 ± 15.55	120.19 ± 14.80	0.589	82.38 ± 10.391	**<0.001**
Hip circumference (cm)	123.25 ± 14.25	122.11 ± 11.89	0.757	99.59 ± 6.829	**<0.001**
WHR	0.96 ± 0.08	0.99 ± 0.08	0.237	0.82 ± 0.062	**<0.001**
Content of fat mass (kg)	51.52 ± 16.76	53.08 ± 16.70	0.740	17.98 ± 6.46	**<0.001**
Content of fat mass (%)	44.93 ± 7.82	45.45 ± 7.40	0.807	24.54 ± 6.40	**<0.001**
Content of lean mass (kg)	61.19 ± 11.84	62.11 ± 11.99	0.783	54.22 ± 10.94	**0.003**
Content of lean mass (%)	55.08 ± 7.82	54.49 ± 7.28	0.781	75.46 ± 6.40	**<0.001**

Values are presented as means ± SD; *n*—number of people; *p* *—level of statistical significance upon comparison of NGT vs. AGT; *p* **—level of statistical significance upon comparison between investigated group and control group; BMI—Body Mass Index; WHR—Waist–Hip Ratio.

**Table 2:** Comparison of selected parameters of glucose and lipid metabolism, blood pressure and omentin-1 concentration in the study groups (without subgrouping).

Parameter	Investigated Group Obesity (*n* = 51)	Control Group no Obesity (*n* = 37)	*p*
Fasting glycaemia (mmol/L)	5.65 (5.15; 6.12)	4.99 (4.55; 5.38)	**<0.001**
Fasting insulinaemia (mU/mL)	12.3 (8.95; 18.45)	6.4 (4.1; 8.4)	**<0.001**
HOMA-IR	3.12 (2.22; 4.38)	1.37 (0.91; 1.92)	**<0.001**
Total cholesterol concentration (mmol/L)	5.58 ± 1.35	5.33 ± 0.86	0.339
LDL concentration (mmol/L)	3.67 (2.83; 4.38)	3.31 (2.66; 3.90)	0.124
HDL concentration (mmol/L)	1.11 (0.94; 1.30)	1.78 (1.34; 1.94)	**<0.001**
Triglyceridaemia (mmol/L)	1.89 (1.38; 2.67)	1.06 (0.78; 1.38)	**<0.001**
Systolic blood pressure (mm Hg)	140 (137.5; 154)	125 (106; 132)	**<0.001**
Diastolic blood pressure (mm Hg)	90 (80; 100)	80 (75; 91)	**<0.001**
Omentin-1 concentration (ng/mL)	234.36 ± 104.06	286.97 ± 113.23	**0.027**

Values are presented as means ± SD or as medians and 25%; 75% percentiles, *n*—number of people; *p*—level of statistical significance; HOMA-IR—Homeostatic Model Assessment–Insulin Resistance; LDL—Low Density Lipoprotein Cholesterol; HDL—High Density Lipoprotein Cholesterol.

**Table 3:** Comparison of selected parameters of glucose and lipid metabolism, blood pressure and omentin-1 concentration in subjects with central obesity by subgroup (with normal or impaired glucose tolerance).

Parameter	Investigated Group Obesity (*n* = 51)		*p*
	Normal Glucose Tolerance (*n*= 24)	Abnormal Glucose Tolerance (*n* = 27)	
**Fasting glycaemia (mmol/L)**	5.20 ± 0.42	6.21 ± 0.93	**<0.001**
Glycaemia at 120′ of OGTT (mmol/L)	5.72 ± 1.20	9.49 ± 2.26	**<0.001**
Fasting insulinaemia (mU/mL)	9.90 (7.78; 14.38)	15.50 (10.00; 20.45)	**0.014**
HOMA-IR	2.33 (1.80; 3.45)	4.00 (2.72; 5.63)	**<0.001**
Total cholesterol concentration (mmol/L)	5.91 ± 1.52	5.27 ± 1.13	0.091
LDL concentration (mmol/L)	4.10 (2.92; 4.59)	3.42 (2.76; 4.17)	0.264
HDL concentration (mmol/L)	1.14 (0.97; 1.36)	1.07 (0.89; 1.24)	0.555
Triglyceridaemia (mmol/L)	1.80 (1.31; 2.81)	1.92 (1.46; 2.47)	0.757
Systolic blood pressure (mm Hg)	140.00 (135.00; 146.25)	150.00 (140.00; 165.00)	**0.038**
Diastolic blood pressure (mm Hg)	90.00 (80.00; 91.25)	90.00 (83.00; 100.00)	0.168
Omentin-1 concentration (ng/mL)	262.77 ± 122.54	209.10 ± 78.30	0.065

Values are presented as means ± SD or as medians and 25%; 75% percentiles, *n*—number of people; *p*—level of statistical significance; OGTT—Oral Glucose Tolerance Test; HOMA-IR—Homeostatic Model Assessment–Insulin Resistance; LDL—Low Density Lipoprotein Cholesterol; HDL—High Density Lipoprotein Cholesterol.

**Table 4:** Comparison of omentin-1 concentrations across gender groups.

	Studied Population (*n* = 88)					
	Women with Obesity (*n* = 27)	Men with Obesity (*n* = 24)	*p* *	Women without Obesity (*n* = 20)	Men without Obesity (*n* = 17)	*p* **
Omentin-1 concentration [ng/mL]	220.87 (165.35; 291.31)	224.43 65.45 303.37	0.923	293.24 (74.23; 346.36)	281.01 (214.55; 336.39)	0.902
	**Women with obesity (*n* = 27)**	**Women without obesity (*n* = 20)**	***p*** **#**	**Men** **with obesity** **(*n* = 24)**	**Men without obesity** **(*n* = 17)**	***p*** **##**
Omentin-1 concentration [ng/mL]	220.87 (165.35; 291.30)	293.24 (74.24; 346.36)	0.121	224.43 (65.45; 303.37)	281.01 (214.55; 336.39)	**0.051**

Values are presented as medians and 25%; 75% percentiles, *n*—number of people; *p* *—level of statistical significance upon comparison of women with obesity vs. men with obesity, *p* **– level of statistical significance upon comparison of women without obesity vs. men without obesity, *p* #—level of statistical significance upon comparison of women with obesity vs. women without obesity, *p* ##—level of statistical significance upon comparison of men with obesity vs. men without obesity.

**Table 5:** Comparison of omentin-1 concentrations in obese subjects with normal glucose tolerance (NGT) and abnormal glucose tolerance (AGT), including gender.

	Investigated Group Obesity (*n* = 51)					
	Normal Glucose Tolerance (*n*= 24)			Abnormal Glucose Tolerance (*n* = 27)		
	Women (*n* = 15)	Men (*n* = 9)	*p* *	Women (*n* = 12)	Men (*n* = 15)	*p* **
Omentin-1 concentration [ng/mL]	218.44 (155.30; 331.36)	301.98 (222.41; 348.72)	**0.019**	223.66 (179.31; 263.77)	214.55 (155.23; 236.88)	0.527
	**Women with obesity (*n* = 27)**			**Men with obesity (*n* = 24)**		
	Normal glucose tolerance (*n* = 15)	Abnormal glucose tolerance (*n* = 12)	*p* ^#^	Normal glucose tolerance (*n* = 9)	Abnormal glucose tolerance (*n* = 15)	*p* ^##^
Omentin-1 concentration [ng/mL]	218.44 (155.30; 331.36)	223.66 (179.31; 263.77)	0.867	301.98 (222.41; 348.72)	214.55 (155.23; 236.88)	**0.035**

Values are presented as medians and 25%; 75% percentiles, *n*—number of people; *p* *—level of statistical significance upon comparison of women with NGT vs. men with NGT, *p* **—level of statistical significance upon comparison of women with AGT vs. men with AGT, *p* ^#^—level of statistical significance upon comparison of women with NGT vs. women with AGT, *p* ^##^—level of statistical significance upon comparison of men with NGT vs. men with AGT.

**Table 6:** Correlations between omentin-1 concentration and anthropometric and metabolic parameters in the investigated and control groups, including gender.

Investigated Group Obesity (*n* = 51)						
Parameter	OMENTIN-1 (ng/mL)					
	Women		Men		Population	
	r_p_/r_s_	*p*	r_p_/r_s_	*p*	r_p_/r_s_	*p*
Body mass (kg)	**−0.453**	**0.018**	−0.089	0.679	−0.244	0.083
BMI (kg/m^2^)	**−0.404**	**0.036**	−0.065	0.761	−0.233	0.100
Waist circumference (cm)	−0.157	0.432	0.056	0.794	−0.061	0.671
WHR	0.334	0.088	0.298	0.157	0.189	0.183
Content of fat mass (%)	−0.372	0.056	−0.037	0.862	−0.201	0.157
Fasting glycaemia (mmol/L)	0.013	0.948	−0.359	0.084	−0.158	0.268
Fasting insulinaemia (µU/L)	−0.275	0.164	0.089	0.676	−0.145	0.310
HOMA-IR	−0.213	0.285	−0.052	0.806	−0.190	0.179
Glycaemia at 120′ of OGTT (mmol/L)	0.109	0.588	**−0.424**	**0.038**	−0.109	0.445
Total cholesterol concentration (mmol/L)	0.077	0.701	0.012	0.953	0.019	0.891
LDL concentration (mmol/L)	0.116	0.563	0.034	0.873	0.063	0.657
HDL concentration (mmol/L)	−0.037	0.852	−0.132	0.536	0.197	0.441
Triglyceridaemia (mmol/L)	0.344	0.078	0.104	0.628	0.217	0.126
SBP (mm Hg)	−0.184	0.358	−0.219	0.303	−0.200	0.158
DBP (mm Hg)	−0.081	0.686	−0.302	0.151	−0.187	0.189
**Control group** **No obesity** **(*n* = 37)**						
**Parameter**	OMENTIN-1 (ng/mL)					
	Women		Men		Population	
	r_p_/r_s_	*p*	r_p_/r_s_	*p*	r_p_/r_s_	*p*
Body mass (kg)	0.361	0.118	0.276	0.283	0.188	0.263
BMI (kg/m^2^)	0.242	0.304	0.351	0.167	0.292	0.079
Waist circumference (cm)	0.377	0.101	0.389	0.122	0.197	0.241
WHR	0.116	0.626	0.246	0.340	0.084	0.619
Content of fat mass (%)	0.200	0.397	0.067	0.797	0.184	0.274
Fasting glycaemia (mmol/L)	−0.029	0.901	−0.024	0.925	0.052	0.758
Fasting insulinaemia (µU/L)	**0.445**	**0.049**	0.230	0.373	**0.356**	**0.030**
HOMA-IR	0.390	0.089	0.240	0.353	0.321	0.052
Total cholesterol concentration (mmol/L)	0.158	0.504	−0.111	0.669	0.046	0.785
LDL concentration (mmol/L)	0.176	0.455	−0.220	0.394	0.034	0.840
HDL concentration (mmol/L)	−0.135	0.568	0.150	0.563	0.031	0.852
Triglyceridaemia (mmol/L)	0.387	0.092	−0.168	0.519	0.110	0.514
SBP (mm Hg)	0.019	0.937	0.042	0.872	−0.191	0.502
DBP (mm Hg)	0.192	0.415	0.156	0.548	−0.121	0.210

r_p_/r_s_—coefficient of Pearson or Spearman (for, respectively, parametric or nonparametric date distributions), *p*—level of statistical significance; BMI—Body Mass Index; WHR—Waist–Hip Ratio; HOMA-IR—Homeostatic Model Assessment–Insulin Resistance; OGTT—Oral Glucose Tolerance Test; LDL—Low Density Lipoprotein Cholesterol; HDL—High Density Lipoprotein Cholesterol; SBP—Systolic Blood Pressure; DBP—Diastolic Blood Pressure.

**Table 7:** Comparison of the two models explaining variations of omentin-1 concentrations before and after adding the fourth variable (gender status) to the basic model, which includes results in oral glucose test, WHR and fat mass [kg] in the entire examined population (*n* = 88).

Statistical Parameters	Model Includes Three Variables: Results in OGTT, WHR and Fat Mass [kg]	Extended Basic Model with a New Variable (Gender Status)
R^2^adj.	0.102	0.137
F	4.305	4.467
*p*	**0.007**	**0.003**

R^2^adj.—adjusted R-squared; F—F statistic; *p*—level of statistical significance.

**Table 8:** Factors associated with high omentin-1 concentrations (above the median value) in male (*n* = 41) and female populations (*n* = 47), based on the results of logistic regression models.

Factors	b	SE (b)	Wald Statistics	OR (95% CI)	*p*
**MEN** (***n* = 41**) *R^2^ Nagelkerke = 0.169; LRT = -25.535 (p = 0.019); the Hosmer–Lemeshow statistic = 4.991 (p = 0.800)*					
Constant	3.044	1.495	4.145	20.987 (1.120–393.148)	**0.042**
Body mass (kg)	−0.030	0.014	4.606	0.971 (0.945–0.997)	**0.032**
**WOMEN** (***n* = 47**) *R^2^ Nagelkerke = 0.120; LRT = -30.270 (p = 0.035); the Hosmer–Lemeshow statistic = 9.658 (p = 0.209)*					
Constant	2.252	1.105	4.156	9.504 (1.091–82.809)	**0.041**
Body mass (kg)	−0.025	0.012	3.987	0.976 (0.952–0.999)	**0.046**

LRT—the likelihood ratio chi-square test; b—regression coefficient in Wald-statistic; SE (b)—standard error in Wald-statistic; OR—Odds ratio; *p*—level of statistical significance.

## Data Availability

The data presented in this study are available on request from the corresponding author. The data are not publicly available due to privacy restrictions.

## References

[1] Alberti K.G., Eckel R.H., Grundy S.M., Zimmet P.Z., Cleeman J.I., Donato K.A., Fruchart J.C., James W.T., Loria C.M., Smith S.C. (2009). Harmonizing the metabolic syndrome. A joint interim statement of the International Diabetes Federation Task Force on Epidemiology and Prevention; National Heart, Lung, and Blood Institute; American Heart Association; World Heart Federation; International Atherosclerosis Society; and International Association for the Study of Obesity. Circulation.

[2] Bays H.E. (2011). Adiposopathy is “sick fat” a cardiovascular disease?. J. Am. Coll. Cardiol..

[3] Pérez-Martínez P., Mikhailidis D.P., Athyros V.G., Bullo M., Couture P., Covas M.I., de Koning L., Delgado-Lista J., Díaz-López A., Drevon C.A. (2017). Lifestyle recommendations for the prevention and management of metabolic syndrome: An international panel recommendation. Nutr. Rev..

[4] (2022). WHO European Regional Obesity Report 2022.

[5] Papaetis G.S., Papakyriakou P., Panagiotou T.N. (2015). Central obesity, type 2 diabetes and insulin: Exploring a pathway full of thorns. Arch. Med. Sci..

[6] Jung H.N., Jung C.H. (2021). The role of anti-inflammatory adipokines in cardiometabolic disorders: Moving beyond adiponectin. Int. J. Mol. Sci..

[7] Arab A., Moosavian S.P., Hadi A., Karimi E., Nasirian M. (2020). The association between serum omentin level and bodyweight: A systematic review and meta-analysis of observational studies. Clin. Nutr. ESPEN.

[8] Askin L., Duman H., Ozyıldız A., Tanriverdi O., Turkmen S. (2020). Association between omentin-1 and coronary artery disease: Pathogenesis and clinical research. Curr. Cardiol. Rev..

[9] Watanabe T., Watanabe-Kominato K., Takahashi Y., Kojima M., Watanabe R. (2011). Adipose tissue-derived omentin-1 function and regulation. Compr. Physiol..

[10] Landecho M.F., Tuero C., Valentí V., Bilbao I., de la Higuera M., Frühbeck G. (2019). Relevance of leptin and other adipokines in obesity-associated cardiovascular risk. Nutrients.

[11] Zhao A., Xiao H., Zhu Y., Liu S., Zhang S., Yang Z., Du L., Li X., Niu X., Wang C. (2022). Omentin-1: A newly discovered warrior against metabolic related diseases. Expert Opin. Ther. Targets.

[12] Auguet T., Quintero Y., Riesco D., Morancho B., Terra X., Crescenti A., Broch M., Aguilar C., Olona M., Porras J.A. (2011). New adipokines vaspin and omentin. Circulating levels and gene expression in adipose tissue from morbidly obese women. BMC Med. Genet..

[13] Alizadeh S., Mirzaei K., Mohammadi C., Keshavarz S.A., Maghbooli Z. (2017). Circulating omentin-1 might be associated with metabolic health status in different phenotypes of body size. Arch. Endocrinol. Metab..

[14] Orlik B., Madej P., Owczarek A., Skałba P., Chudek J., Olszanecka-Glinianowicz M. (2014). Plasma omentin and adiponectin levels as markers of adipose tissue dysfunction in normal weight and obese women with polycystic ovary syndrome. Clin. Endocrinol..

[15] Olszanecka-Glinianowicz M., Madej P., Owczarek A., Chudek J., Skałba P. (2015). Circulating anti-Müllerian hormone levels in relation to nutritional status and selected adipokines levels in polycystic ovary syndrome. Clin. Endocrinol..

[16] World Health Organization, International Diabetes Federation (1999). Definition, Diagnosis and Classification of Diabetes Mellitus and Its Complications.

[17] Williams B., Mancia G., Spiering W., Agabiti Rosei E., Azizi M., Burnier M., Clement D.L., Coca A., de Simone G., Dominiczak A. (2018). 2018 ESC/ESH Guidelines for the management of arterial hypertension: The Task Force for the management of arterial hypertension of the European Society of Cardiology (ESC) and the European Society of Hypertension (ESH). Eur. Heart J..

[18] Friedewald W.T., Levy R.I., Fredrickson D.S. (1972). Estimation of the concentration of low-density lipoprotein cholesterol in plasma, without use of the preparative ultracentrifuge. Clin. Chem..

[19] Hosmer D.W., Lemeshow S.A., Sturdivant R.X. (2013). Applied Logistic Regression.

[20] Zhou J.Y., Chan L., Zhou S.W. (2014). Omentin: Linking metabolic syndrome and cardiovascular disease. Curr. Vasc. Pharmacol..

[21] Moreno-Navarrete J.M., Ortega F., Castro A., Sabater M., Ricart W., Fernández-Real J.M. (2012). Circulating Omentin as a Novel Biomarker of Endothelial Dysfunction. Obesity.

[22] Moreno-Navarrete J.M., Catalán V., Ortega F., Gómez-Ambrosi J., Ricart W., Frühbeck G., Fernández-Real J.M. (2010). Circulating omentin concentration increases after weight loss. Nutr. Metab..

[23] Lesná J., Tichá A., Hyšpler R., Musil F., Bláha V., Sobotka L., Zadák Z., Šmahelová A. (2015). Omentin-1 plasma levels and cholesterol metabolism in obese patients with diabetes mellitus type 1: Impact of weight reduction. Nutr. Diabetes.

[24] Arslan I., Ulas T., Karakas E.Y., Demir M., Eren M.A., Torun A., Sabuncu T. (2017). Comparative effectiveness of diet alone and diet plus metformin treatment on omentin levels in type 2 diabetes patients with nonalcoholic fatty liver disease: A prospective randomized trial. Period. Biol..

[25] Antonio de Luis D., Izaola O., Primo D., Aller R. (2018). Modifications of serum levels of omentin-1 and other cardiovascular risk factors following weight loss secondary to a Mediterranean hypocaloric diet. Clin. Nutr..

[26] Derosa G., Fogari E., D’Angelo A., Bianchi L., Bonaventura A., Romano D., Maffioli P. (2013). Adipocytokine levels in obese and non-obese subjects: An observational study. Inflammation.

[27] Pan X., Kaminga A., Wen S., Acheampong K., Liu A. (2019). Omentin-1 in diabetes mellitus: A systematic review and meta-analysis. PLoS ONE.

[28] Du Y., Ji Q., Cai L., Huang F., Lai Y., Liu Y., Yu J., Han B., Zhu E., Zhang J. (2016). Association between omentin-1 expression in human epicardial adipose tissue and coronary atherosclerosis. Cardiovasc. Diabetol..

[29] Watanabe K., Watanabe R., Konii H., Shirai R., Sato K., Matsuyama T.A., Ishibashi-Ueda H., Koba S., Kobayashi Y., Hirano T. (2016). Counteractive effects of omentin-1 against atherogenesis. Cardiovasc. Res..

[30] Escoté X., Gómez-Zorita S., López-Yoldi M., Milton-Laskibar I., Fernández-Quintela A., Martínez J.A., Moreno-Aliaga M.J., Portillo M.P. (2017). Role of omentin, vaspin, cardiotrophin-1, TWEAK and NOV/CCN3 in obesity and diabetes development. Int. J. Mol. Sci..

[31] Pan H.Y., Guo L., Li Q. (2010). Changes of serum omentin-1 levels in normal subjects and in patients with impaired glucose regulation and with newly diagnosed and untreated type 2 diabetes. Diabetes Res. Clin. Pract..

[32] Cai R.C., Wei L., Di J.Z., Yu H.Y., Bao Y.Q., Jia W.P. (2009). Expression of omentin-1 in adipose tissues in obese and type 2 diabetic patients. Zhonghua Yi Xue Za Zhi.

[33] Halabis M., Dziedzic M., Warchulinska J., Kazanowska-Bystryk I., Solski J. (2015). Omentin—A new adipokine with many roles to play. Curr. Issues Pharm. Med. Sci..

[34] Flehmig G., Scholz M., Klöting N., Fasshauer M., Tönjes A., Stumvoll M., Youn B.S., Blüher M. (2014). Identification of adipokine clusters related to parameters of fat mass, insulin sensitivity and inflammation. PLoS ONE.

[35] Elsaid N.H., Sadik N.A., Ahmed N.R., Fayez S.E., Mohammed N.A. (2018). Serum omentin-1 levels in type 2 diabetic obese women in relation to glycemic control, insulin resistance and metabolic parameters. J. Clin. Transl. Endocrinol..

[36] Komosińska-Vassev K., Olczyk P., Kuźnik-Trocha K., Jura-Półtorak A., Derkacz A., Purchałka M., Telega A., Olczyk K. (2019). Circulating C1q/TNF-related protein 3, omentin-1 and NGAL in obese patients with type 2 diabetes during insulin therapy. J. Clin. Med..

[37] Jaikanth C., Gurumurthy P., Cherian K.M., Indhumathi T. (2013). Emergence of omentin as a pleiotropic adipocytokine. Exp. Clin. Endocrinol. Diabetes.

[38] Katsi V., Vamvakou G., Lekakis J., Tousoulis D., Stefanadis C., Makris T., Kallikazaros I. (2014). Omentin, fat and heart: Classical music with new instruments. Heart Lung Circ..

[39] Sitticharoon C., Nway N.C., Chatree S., Churintaraphan M., Boonpuan P., Maikaew P. (2014). Interactions between adiponectin, visfatin, and omentin in subcutaneous and visceral adipose tissues and serum, and correlations with clinical and peripheral metabolic factors. Peptides.

[40] Tan B., Adya R., Randeva H. (2010). Omentin: A novel link between inflammation, diabesity, and cardiovascular disease. Trends Cardiovasc. Med..

[41] Urbanová M., Dostálová I., Trachta P., Drápalová J., Kaválková P., Haluzíková D., Matoulek M., Lacinová Z., Mráz M., Kasalický M. (2014). Serum concentrations and subcutaneous adipose tissue mRNA expression of omentin in morbid obesity and type 2 diabetes mellitus: The effect of very-low-calorie diet, physical activity and laparoscopic sleeve gastrectomy. Physiol. Res..

[42] Cătoi A.F., Suciu Ş., Pârvu A.E., Copăescu C., Galea R.F., Buzoianu A.D. (2014). Increased chemerin and decreased omentin-1 levels in morbidly obese patients are correlated with insulin resistance, oxidative stress and chronic inflammation. Clujul Med..

[43] Hossein-Nezhad A., Mirzaei K., Alatab S., Ahmadivand Z., Najmafshar A. (2014). Circulating omentin-1 in obesity and metabolic syndrome status compared to control subjects. Endocrinol. Metab. Syndr..

[44] Onur I., Oz F., Yildiz S., Oflaz H., Sigirci S., Elitok A., Pilten S., Karaayvaz E.B., Cizgici A.Y., Kaya M.G. (2014). Serum omentin 1 level is associated with coronary artery disease and its severity in postmenopausal women. Angiology.

[45] Xu T., Zuo P., Cao L., Gao Z., Ke K. (2018). Omentin-1 is associated with carotid plaque instability among ischemic stroke patients. J. Atheroscler. Thromb..

[46] Niersmann C., Carstensen-Kirberg M., Maalmi H., Holleczek B., Roden M., Brenner H., Herder C., Schöttker B. (2020). Higher circulating omentin is associated with increased risk of primary cardiovascular events in individuals with diabetes. Diabetologia.

[47] Wittenbecher C., Menzel J., Carstensen-Kirberg M., Biemann R., di Giuseppe R., Fritsche A., Isermann B., Herder C., Aleksandrova K., Boeing H. (2016). Omentin-1, adiponectin, and the risk of developing type 2 diabetes. Diabetes Care.

[48] Shibata R., Ouchi N., Kikuchi R., Takahashi R., Takeshita K., Kataoka Y., Ohashi K., Ikeda N., Kihara S., Murohara T. (2011). Circulating omentin is associated with coronary artery disease in men. Atherosclerosis.

[49] Lin S., Li X., Zhang J., Zhang Y. (2021). Omentin-1: Protective impact on ischemic stroke via ameliorating atherosclerosis. Clin. Chim. Acta.

[50] Çimen A.R., Cerit E.T., Iyidir O.T., Karakus R., Uyar B.B., Toruner F.B., Cakir N., Arslan M. (2017). Serum omentin-1 levels and endothelial dysfunction in obesity. Acta Endocrinol..

[51] Ohashi K., Shibata R., Murohara T., Ouchi N. (2014). Role of anti-inflammatory adipokines in obesity-related diseases. Trends Endocrinol. Metab..

[52] Aliasghari F., Izadi A., Jabbari M. (2018). Are vaspin and omentin-1 related to insulin resistance, blood pressure and inflammation in NAFLD patients?. J. Med. Biochem..

[53] de Souza Batista C.M., Yang R.Z., Lee M.J., Glynn N., Yu D.Z., Pray J., Ndubuizu K., Patil S., Schwartz A., Kligman M. (2007). Omentin plasma levels and gene expression are decreased in obesity. Diabetes.

[54] Babaei P., Damirchi A., Ghouroughchi A. (2016). The effect of estrogen on visceral fat, serum omentin-1 and insulin resistance in ovariectomized rats. J. Ardabil. Univ. Med. Sci..

[55] Borowski A., Siemińska L. (2020). Serum omentin levels in patients with prostate cancer and associations with sex steroids and metabolic syndrome. J. Clin. Med..

